# Development and Psychometric Evaluation of Healthcare Access Measures among Women with Ovarian Cancer

**DOI:** 10.3390/cancers14246266

**Published:** 2022-12-19

**Authors:** Tomi Akinyemiju, Ashwini Joshi, April Deveaux, Lauren E. Wilson, Dandan Chen, Clare Meernik, Malcolm Bevel, Jen Gathings, Laura Fish, Nadine Barrett, Valarie Worthy, Xiomara Boyce, Keshia Martin, Corre Robinson, Maria Pisu, Margaret Liang, Arnold Potosky, Bin Huang, Kevin Ward, Maria J. Schymura, Andrew Berchuck, Bryce B. Reeve

**Affiliations:** 1 Department of Population Health Sciences, Duke University School of Medicine, Durham, NC 27708, USA; 2 Duke Cancer Institute, Duke University School of Medicine, Durham, NC 27710, USA; 3 ETR Services, Durham, NC 27705, USA; 4 Division of Preventive Medicine, O’Neal Comprehensive Cancer Center, University of Alabama at Birmingham, Birmingham, AL 35233, USA; 5 Cancer Prevention and Control Program Lombardi Comprehensive Cancer Center, Georgetown University, Washington, DC 20007, USA; 6 Kentucky Cancer Registry, University of Kentucky, Lexington, KY 40506, USA; 7 Georgia Cancer Registry, Emory University, Atlanta, GA 30322, USA; 8 New York State Cancer Registry, New York State Department of Health, Albany, NY 12203, USA; 9 Division of Gynecologic Oncology, Duke Cancer Institute, Duke University School of Medicine, Durham, NC 27710, USA

**Keywords:** healthcare access, qualitative study, concept elicitation, cancer, racial disparities, psychometrics

## Abstract

**Simple Summary:**

The Ovarian Cancer Epidemiology, Healthcare Access and Disparities study aims to characterize healthcare access (HCA) across five specific dimensions—Availability, Affordability, Accessibility, Accommodation and Acceptability—among Black, Hispanic and White patients with ovarian cancer (OC) to evaluate the impact of HCA on quality of treatment, supportive care and survival, and explore biological mechanisms that may contribute to OC disparities. Currently, there are no validated instruments for empirically measuring all HCA dimensions. To characterize HCA among diverse cancer survivors, there is a need to develop reliable, content-relevant, and comprehensive measures that have cross-cultural applicability. Thus, the goal of the present study was to: (1) develop a comprehensive survey instrument for HCA, guided by the Penchansky and Thomas framework; and (2) evaluate the factor structure, reliability, and psychometric properties of two domains (Accommodation and Acceptability) that are only estimable from patient-reported survey data.

**Abstract: Introduction:**

There are no validated instruments for measuring healthcare access (HCA). This study aimed to develop a cohesive HCA instrument for cancer survivors and evaluate the factor structure, reliability, and psychometric properties of two HCA domains—Acceptability and Accommodation—that require patient-reported survey data. **Methods:** This study reports data from three general methodological approaches: (1) concept elicitation using focus groups with 32 cancer survivors (63% Black, 18% Hispanic) to inform the development of new HCA survey items; (2) refining the new survey items using cognitive interviews with seven ovarian cancer survivors (n = 3 Black, n = 1 Hispanic) and pilot testing with 54 ovarian cancer survivors (74% White, 14% Black); and (3) psychometric evaluation of the Acceptability and Accommodation items among 333 ovarian cancer survivors (82% White, 13% Black). Multiple model structures were assessed for each HCA dimension using confirmatory factor analysis methods, and composite reliability was estimated for selected models. **Results:** In focus group discussions, cancer survivors expressed challenges navigating cancer treatment across multiple HCA domains, with the Acceptability domain (quality of patient–provider interaction) being the most salient across all racial groups. Lack of empathy, compassion, and poor communication overshadowed positive aspects of providers’ specialty, experience, or reputation. Cognitive interviews and pilot testing of new HCA survey items helped to clarify the language of specific items and refine the recruitment and consent process for implementation of the survey. In psychometric evaluation, the Accommodation domain (convenience and organization of services) was best accounted for by a two-factor structure: satisfaction with care and access to support services (comparative fit index (CFI) = 0.99). For the Acceptability domain, all fit indices supported the retention of a five-factor higher-order model (CFI = 0.96). Composite reliability estimates were >0.80 for all latent factors in the two-factor Accommodation model and the higher-order Acceptability model. **Conclusions:** Empirical evidence supports the utility of standardized measures of Accommodation and Acceptability using self-reported survey items, which will contribute to the better characterization of HCA dimensions among diverse cancer survivors.

## 1. Introduction

Several proposed theoretical frameworks have defined the complex nature of healthcare access (HCA) [1,2,3], although the Penchansky and Thomas framework remains the most highly regarded and comprehensive [3]. This framework defines HCA as five separate but related dimensions: Affordability (ability to pay for care), Availability (quality and volume of healthcare services), Accessibility (location of services in relation to patient), Accommodation (organization of services and convenience for patients), and Acceptability (quality of patient–provider interaction) [3,4]. Although developed in the context of primary care, it provides a strong theoretical foundation for evaluating the interaction of multiple factors operating at the patient, provider, hospital, and healthcare system levels to impact the quality of cancer care.

Despite extensive study, there are currently no validated instruments for empirically measuring all HCA dimensions. While certain dimensions (e.g., Affordability) have established measures [5,6,7], others, including the Accommodation and Acceptability domains, rely on self-reported data and currently lack standardized measures [3,4]. Furthermore, patient-reported experiences with HCA have been understudied in racially and ethnically diverse populations, contributing to significant knowledge gaps across racial and ethnic groups in: the relative importance of HCA dimensions; the interaction between multiple HCA dimensions; and the interaction between HCA dimensions and patient-level factors (e.g., language, health literacy) that may affect disparities in quality of cancer treatment. To empirically characterize HCA among diverse cancer survivors (starting from the time of diagnosis until the end of life), there is a need to develop reliable, content-relevant, and comprehensive measures that have cross-cultural applicability.

Thus, the goal of the present study was to: (1) develop a comprehensive survey instrument for HCA, guided by the Penchansky and Thomas framework; and (2) evaluate the factor structure, reliability, and psychometric properties of two domains (Accommodation and Acceptability) that are only estimable from patient-reported survey data.

## 2. Methods

**Figure 1:**
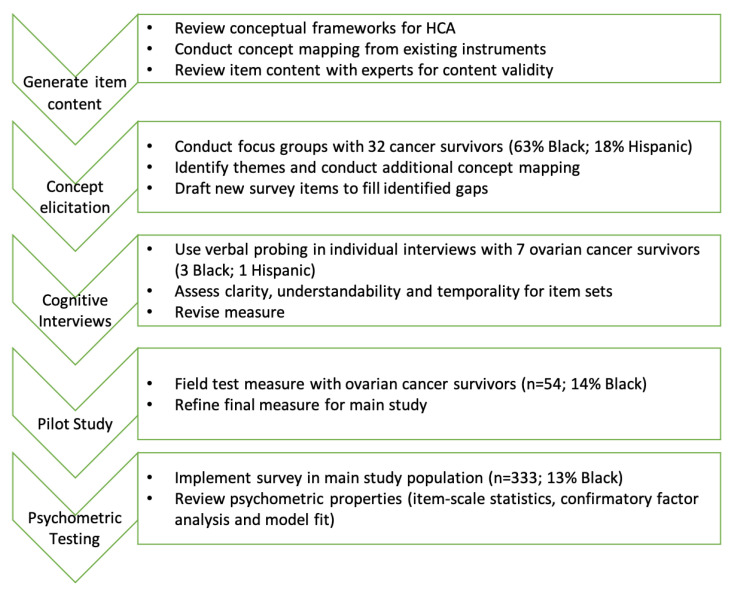
Steps to develop HCA Accommodation and Acceptability Measures for diverse cancer survivors.

Figure 1 summarizes the measurement development steps used in this study. Embedded within the larger population-based ovarian cancer epidemiology, healthcare access and disparities (ORCHiD) study and following standard approaches for measurement development, we reviewed foundational papers on HCA domains for concept mapping (details previously published [8]); conducted focus groups for additional concept mapping and drafting of new HCA survey items to fill identified gaps; conducted cognitive interviews and pilot testing to revise and refine final HCA items; and examined the psychometric properties of the Accommodation and Acceptability items.

### 2.1. A. Concept Elicitation

*Participants and Settings:* A total of 32 (63% Black, 18% Hispanic) cancer survivors were invited to participate in focus groups to understand their treatment journey and experience accessing care. We partnered with cancer support group organizations for the recruitment of women with a history of any cancer. An introductory letter was sent to each support group organization explaining the study rationale, methodology and requirements and requesting permission to recruit support group members for the study. Interested participants contacted the study team to sign up for focus group sessions. Due to COVID-19 restrictions, focus group discussions were conducted virtually in groups of three to six cancer survivors. Each participant was compensated $25 for their time and effort.

*Procedures and Data Collection:* Focus group sessions were moderated by an experienced facilitator and at least one note-taker, using a structured topic guide (Supplemental Material). The topic guide was developed to facilitate open-ended discussion about participants’ cancer treatment and experiences accessing care, with probing questions addressing each of the five HCA dimensions. Sessions were audio-taped and ranged from 43 min to 92 min (median: 75 min); six groups were conducted in English, and one was conducted in Spanish. Audio files were collated and transcribed verbatim by a professional service. The focus group with Spanish-speaking survivors was first transcribed into Spanish and then translated into English, and the English version of the transcript was used for coding purposes.

*Qualitative Analysis:* Focus group data were coded and analyzed using NVivo 12. Following established methodology, peer debriefing was used to evaluate the credibility and completeness of the analysis [9]. This strategy involves sharing the coding scheme, analytic memos, and next-to-final drafts with knowledgeable colleagues to discuss the results and alternate interpretations of the data. Guided by the Penchansky and Thomas’s HCA framework, a coding scheme including *a priori* HCA domains was drafted and shared with the full research team to assess content validity. A coder then used a query function to generate results for each of the five HCA dimensions and key topics, such as perceptions of access to care, choice in care, and challenges encountered. [3,4]. The full transcripts were first reviewed in their entirety to gain familiarity with the data. Initial readings of the transcript suggested the existence of several emergent codes due to their frequency in the data, and they were defined as facilitators and barriers to treatment. Operational definitions and examples are provided in Table 1. To estimate the relative frequency of each HCA dimension, we calculated the total number of mentions (or appearance of codes in transcript conversation) for each dimension divided by the total number of HCA dimensions mentioned.

### 2.2. B. Cognitive Interviews and Pilot Testing

*Cognitive Interviews*: Findings from the concept elicitation (focus groups) informed the development of new survey items to address gaps in existing HCA measures. Cognitive interviews with seven ovarian cancer survivors recruited from the Duke Cancer Institute (n = 3 Black, n = 3 White, n = 1 Hispanic) were then conducted via telephone. Participants provided feedback on each item one at a time, and verbal probing was used to determine the ease of understanding of survey instructions, item wording, and content. Feedback was also solicited to assess the frequency of request for clarification and potential time burden. Cognitive interviews were conducted in two rounds; feedback from the first round was used to revise the survey measures before the second round. Results from both rounds of interviews were used to refine the survey for pilot testing. Participants were compensated with a $25 gift card for their time and effort.

*Pilot Testing*: Pilot testing of the entire survey was conducted among 54 ovarian cancer survivors (74% White, 14% Black) recruited from the Duke Cancer Institute and the University of Alabama at Birmingham Gynecology Oncology clinics. Surveys were administered by telephone, electronically, or paper versions. Participants were compensated with a $25 gift card for their time and effort. This study was approved by Duke University and the participating hospitals’ Institutional Review Boards (IRB) (Pro00101872). The Duke University IRB served as a reviewing IRB for the University of Alabama at Birmingham study protocol under reliance agreement. All participants included provided informed consent.

### 2.3. C. Psychometric Evaluation

*Participants:* Psychometric evaluation of the Accommodation and Acceptability domains was conducted using data from the main ORCHiD study. ORCHiD is a population-based study of ovarian cancer survivors ages 20–75 years at diagnosis recruited from cancer registries in multiple US states (Georgia, Kentucky, New York, Texas, Maryland, and California). Survivors were recruited into the study approximately 12 months post-diagnosis and invited to complete a survey that used multimodal data collection (paper, electronic, or telephone) of demographic characteristics, socioeconomic status, HCA domains, primary cancer treatment, and post-treatment outcomes. Details regarding study methodology and recruitment have been previously described [8]. The current analysis utilizes survey data from 333 (82% White and 13% Black) participants recruited between March 2021 and April 2022.

Study Measures: Self-reported items associated with Accommodation included measures related to the following topics: seeing the same doctor repeatedly, convenience of appointment, satisfaction with care and access to support services. The items were newly developed or derived from the Ambulatory Care Experiences Survey (ACES) [10] or the Medicare Current Beneficiary Survey (MCBS) [11]. Items associated with Acceptability included trust in provider and medical systems, care for emotions, cultural competence, sharing information and interaction with other staff at their provider’s office. These items were newly developed or derived from the Group-Based Medical Mistrust Scale [12], the Patient-Centered Communication in Cancer Care (PCC-Ca)-36 [13], or the Consumer Assessment of Healthcare Providers & Systems (CAPHS) [14]. Details of each dimension and associated survey items are listed in Supplemental Table S1.

*Psychometric Analysis:* Frequencies and percentages were calculated for binary and categorical variables, and medians and interquartile ranges were calculated for continuous variables. We used confirmatory factor analysis (CFA) for each HCA dimension to determine the factor structure. CFA was used because theory and previous research determined the factor structure of each scale [15]. For the Accommodation dimension, we fit a three-factor model informed by the structure of the existing survey measures: (1) access to support services; (2) satisfaction with various aspects of care; and (3) convenience of scheduling appointment and seeing your regular doctor. A one-factor model including all measures was used as an alternative model. For the Acceptability dimension, we fit a five-factor higher-order model which was informed by the structure of the existing survey measures: (1) trust in provider and medical systems; (2) care for emotions; (3) cultural competence; (4) sharing information; and (5) other staff at the doctor’s office. Other alternative models tested included a one-factor model with all measures and a five-factor first-order model. All models were tested using the robust weighted least squares means, and variance adjusted (*WLSMV*) estimator that provides standard errors and fit indices that are robust to the order-categorical nature of the items and can handle missing data [16,17]. To evaluate the quality of measurement models, model fit indices including the root mean square error of approximation (RMSEA; criteria < 0.8) [18], the Tucker–Lewis index (TLI; criteria > 0.90) [19], and the comparative fit index (CFI; criteria > 0.90) [20]. For each HCA dimension, if the model had poor fit or items did not load highly on a factor (>0.30), we tested alternative models with a preference for the more parsimonious model. For the final selected models, we generated composite reliability and average variance extracted (AVE) statistics for each latent factor in the model [21]. Composite reliability values > 0.70 and AVE > 0.50 are considered ideal for multi-item scales [22]. CFA was performed using Mplus 7.31 (Muthen and Muthen, 1998–2013).

## 3. Results

### 3.1. A. Concept Elicitation

A total of 32 cancer survivors participated in seven focus groups; 63% of participants were Black, 19% were White and 18% were Hispanic/Latinx; all Hispanic survivors participated in the Spanish-speaking focus group. Almost one-third of participants (29%) were diagnosed with cancer within the last five years, and 19% were still in active treatment. Cohort descriptive statistics are presented in Supplemental Table S2. A total of 265 HCA domain mentions were captured across the seven focus groups, representing the number of times focus group participants mentioned a theme relating to one of the five HCA dimensions (Table 2). An additional 100 mentions were captured relating to the emergent codes associated with facilitators or barriers to care. Acceptability, Accommodation, Affordability and Availability dimensions were mentioned in all seven focus groups, and Accessibility was mentioned in five focus groups. Two emergent codes were identified: facilitators, including having a support system (59%), and barriers, including fear and emotional awareness (46%) were mentioned in all seven focus groups. Summary descriptions of each dimension and emergent factor are presented below, and illustrative quotes are presented in Supplemental Table S2.

#### 3.1.1. Dimension 1: Acceptability

Acceptability was the most frequently applied code from the HCA framework, comprising more than 40% of all mentions. Survivors extensively discussed how Acceptability impacted their cancer treatment journey, including the importance of bedside manner, patient–provider communication, and comfort with the provider’s reputation or referral from a trusted source. Approximately 75% of all mentions of Acceptability were positive, describing providers with a gentle tone or caring demeanor, those that provided physical touch as a means of comfort, and those that showed empathy, compassion, and respect for their religious beliefs. When issues related to Acceptability were more critical of members of their care team, these comments tended to focus on issues regarding how a diagnosis was delivered, inadequate personnel bedside manner, and poor provider–patient communication. Participants did not emphasize a provider’s race, gender, or qualifications, and instead, they prioritized the quality of the patient–provider interaction as critical to feeling cared for and as a measure of high-quality care.

#### 3.1.2. Dimension 2: Accommodation

The second most frequently mentioned dimension was Accommodation, comprising 20% of all mentions. Several cancer survivors described travel over long distances to cancer treatment facilities and the importance of being able to schedule all appointments, treatment, and bloodwork on the same day. Spanish-speaking survivors noted that translation services and/or bilingual providers were key factors in the quality of their treatment journey, acknowledging the significance of a language barrier.

#### 3.1.3. Dimension 3: Availability

Availability was the third most described HCA domain, comprising 14% of all mentions. Ovarian cancer survivors specifically described the availability of gynecologic oncologists as most important for quality treatment, and geographical barriers to gynecologic oncology consultations were identified as a key challenge for this group of survivors.

#### 3.1.4. Dimension 4: Affordability

Although mentioned less frequently, for 14% of all mentions, Affordability was an important consideration of HCA. While a majority of the survivors in the focus groups had insurance that covered most of their medical costs, several participants reported anxieties and worry regarding the uncertainties about insurance coverage and the adverse impact of high cancer treatment cost on their family’s finances.

#### 3.1.5. Dimension 5: Accessibility

Accessibility was described as a challenge to HCA, with 11% of mentions, especially among survivors in rural areas. Many focus group participants lived near their treatment facility and did not have a significant amount of travel time. However, survivors in rural areas described substantial challenges with finding reliable transportation to providers, and those who relied on county-provided transportation options experienced longer travel times as they had to wait until all fellow travelers completed their visits before returning home.

#### 3.1.6. Emergent Codes: Facilitators and Barriers to Treatment

Two key emergent codes were identified outside of the a priori specified HCA domains, which were categorized as supports/facilitators and challenges/barriers to treatment. Typically, these represented individual characteristics or access to important resources outside of the healthcare setting that may be modifiable and interact with other dimensions of access. Having access to a strong support system (59% of facilitator mentions) supported recovery and positive experiences during one’s treatment journey. Support derived from participation in cancer-related support groups was mentioned most frequently, with many participants noting the importance of connecting with other women with similar experiences. Participants also noted the importance of having family members for support during their treatment journey, including for emotional and tangible support (e.g., information, advocacy, assistance with transportation and appointments, or navigating complicated healthcare systems). The next frequently mentioned code was faith (28% of facilitator mentions)—highlighting the importance of faith and belief in helping survivors persevere along their treatment journey.

Focus group participants also noted barriers that negatively impacted recovery experiences. Fear was the most frequently mentioned (46% of barrier mentions), with participants reporting initial fear in seeking diagnosis and care, fear upon learning of a new diagnosis, and the primacy of fear in outdated cancer narratives. Fear was a salient theme for Hispanic participants who described a fear of having personal or familial immigration status revealed to authorities in healthcare settings and reluctance to share personal information on medical forms “even though they are residents.” Mental and emotional awareness was the next most frequently mentioned by survivors (18% of barrier mentions). Survivors noted the impact of diagnosis on their mental health and cognitive abilities. Some mentioned these effects in terms of shock or being in a fog, likely as part of a trauma response to a new diagnosis. Inadequate social support often exacerbated the fear (15% of barrier mentions). Role conflict (i.e., difficulty negotiating opposing roles, such as being both a cancer survivor and a caregiver for an elderly family member) also emerged as a key barrier (9% of barrier mentions).

### 3.2. B. Cognitive Interviews and Pilot Testing

*Cognitive Interviews*: Survivors participating in cognitive interviews generally reported that the survey was easy to understand. Feedback was incorporated into specific survey items, including defining certain terminology (e.g., PARP inhibitors) and being more specific in the wording of items (e.g., “all of your health care needs” vs. “ovarian cancer treatment needs”).

*Pilot Testing*: After two rounds of cognitive interviews, the survey (assessing all HCA domains) was edited for length, clarity, and content, and then pilot tested. The purpose of the pilot testing was to refine recruitment, consent, and data collection processes for the implementation of the survey in the main study population. Feedback was incorporated from interviewers and pilot testing participants to improve recruitment efficiency (e.g., obtaining participants’ preferred day/time to call back to complete the survey after obtaining informed consent).

### 3.3. C. Psychometric Analysis

A total of 333 women were included in psychometric analysis of Accommodation and Acceptability HCA dimensions. The median age at diagnosis was 60 years (IQR 46–65 years); 81% were non-Hispanic White, 13% were Black/African American, and the remainder of the cohort were of other races, including Asian, Native Hawaiian or Pacific Islander, and Native American or Alaska Native (Supplemental Table S2). Missing data rates for survey measures were low (<5%).

**Figure 2:**
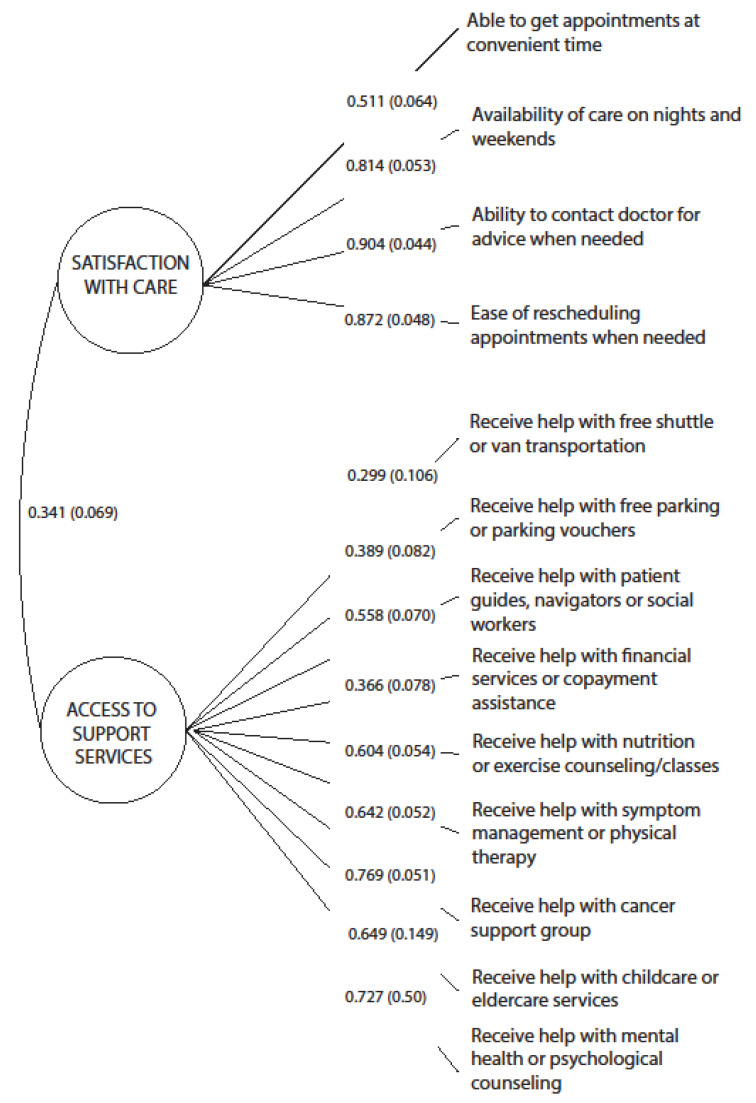
A Two-Factor Model of Accommodation Dimension.

For the Accommodation dimension, the proposed three-factor model fit statistics indicated a satisfactory fit: CFI = 0.98, RMSEA = 0.03 (Table 3); however, the item assessing whether survivors were *able to see the same doctor at most appointments* did not significantly load on its hypothesized factor alongside the item measuring *convenience of scheduling appointments*. As the item *convenience of scheduling appointments* is a similar concept to the latent construct *Satisfaction with Care*, we hypothesized that the *convenience* item might better fit alongside those measures, and the measure of *seeing the same doctor* might instead fit better with measures of Availability or as a stand-alone measure. Therefore, we tested a two-factor model of Accommodation which dropped the item *seeing the same doctor* and shifted the *convenience of scheduling appointments* item to load on *Satisfaction with Care*. This alternative model still showed good fit: CFI = 0.99, RMSEA = 0.03 (Table 3). The factor loadings of all items were statistically significant. Factor loadings linking each variable to their respective factors are presented in Figure 2. The two factors were moderately correlated (*r* = 0.44), indicating that they measure distinct sub-dimensions of Accommodation. Thus, factor scores were derived for *Satisfaction with Care* and *Access to Support Services* based on the two-factor model. The alternative, one-factor model for the Accommodation items had poor fit as measured by the model fit indices compared to the other tested model structures (CFI = 0.78 and RMSEA = 0.10).

**Figure 3:**
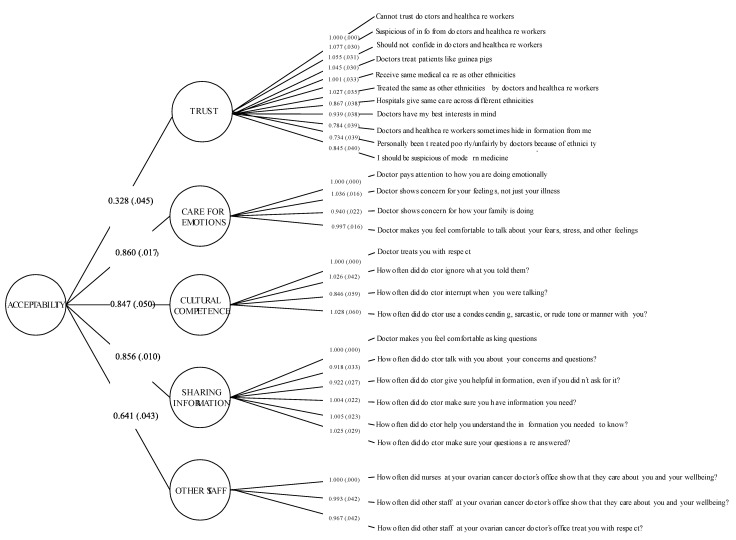
A Five-Factor Higher-Order Model of Acceptability Dimension.

For the Acceptability dimension, model fit indices supported the retention of either: the five-factor first-order model or the five-factor higher-order model (Table 3). Fit statistics were essentially identical between the two models, as correlations were allowed between the latent variables. There is a compelling reason for favoring the higher-order model as the associations among the factors are strong (Supplemental Table S4) (range r = 0.32 to 0.95; highest correlation between the *Sharing Information* and *Cultural Competence* factors). Consequently, the higher-order model was retained (CFI = 0.96, RMSEA = 0.08) and factor scores for Acceptability and its component item groupings were produced based on the multidimensional model. Standardized factor loadings linking each variable to their respective factors are presented in Figure 3.

The composite reliability estimates (Ω) were ≥0.80 for the Accommodation sub-dimensions and ≥0.89 for the Acceptability dimension and all sub-dimensions (Table 4). The high composite reliability and average variance extracted (AVE) for both dimensions provide evidence for the internal consistency of scale items. Although AVE was low for *Access to support services*, it is still acceptable given the high composite reliability [23]. Supplemental Table S4 presents correlations among the Accommodation sub-dimension factor scores (*Satisfaction with Care* and *Access to Support Services*) and the factor scores for the higher order Acceptability dimension and its subfactors (trust, care for emotions, cultural competence, sharing information, and other staff). The moderate correlations indicate that there may be two distinct sub-dimensions of Accommodation (correlation = 0.44), whereas higher correlations for all sub-dimensions of Acceptability, except the trust dimension, indicate the sub-dimensions are more likely part of same latent construct of Acceptability. Considering the low/moderate correlations observed in the Accommodation dimension, this construct will be analyzed as two sub-dimensions (*Satisfaction with Care* and *Access to Support Services*), and Acceptability will be measured using an overall Acceptability score in addition to utilizing factor scores for the individual sub-dimensions of Acceptability.

## 4. Discussion

Understanding the role of multiple dimensions of HCA on cancer outcomes may help inform targeted strategies to mitigate racial disparities; however, the lack of standardized and validated measurement instruments has inhibited rigorous research in this area. It is especially critical to measure distinct HCA dimensions that may independently or jointly impact the quality of cancer care and subsequently, survival outcomes. To our knowledge, this is the first study to report the psychometric properties of the Accommodation and Acceptability HCA dimensions, and to center the experience of Black and Hispanic survivors in developing a cohesive HCA instrument. Results from the concept elicitation interviews highlight that all five theoretical HCA dimensions are important in cancer survivors’ treatment journey, although the Acceptability domain (i.e., quality of patient–provider interaction) was the most salient for cancer survivors. When Acceptability was high, survivors reported being more trusting and comfortable sharing concerns and fears related to their diagnosis, prognosis, and other concerns related to their physical, mental, and emotional health. These findings indicate that the Acceptability dimension can be utilized as a specific and quantifiable measure of patient-centered, high-quality care.

There were racial differences observed in the importance ascribed to certain HCA dimensions by cancer survivors in focus groups. Black and Hispanic survivors were more likely to describe significant challenges with Acceptability, which was consistent with prior studies that have documented that racial minorities are more likely to report a lack of trust in physicians [24,25,26], have negative experiences in healthcare [27], and report perceived or expected discrimination [28,29]. These factors impact the quality of patient–provider interactions, including survivors’ willingness to accept physician recommendations, shared decision making for treatments, and communication [30,31,32,33], indicating that strategies to address this dimension are critical to enhancing HCA and mitigating racial disparities. Accessibility perceptions varied, with survivors residing in small cities or rural areas more likely to experience challenges, including having to rely on public transportation or non-family members for transport to appointments. Hispanic survivors noted challenges including difficulty in accessing language translation services and transportation, though because all Hispanic survivors participated in the Spanish-speaking focus group, it is unclear whether similar barriers would be reported by Hispanic survivors who primarily speak English. Strong support systems, including spouses and children, were key for providing both emotional and tangible support, while faith was a source of spiritual comfort and tangible support. Survivors that lacked support systems experienced the most challenges during their treatment journey, regardless of HCA dimensions, which was consistent with previous studies [34,35,36]. Notably, Hispanic survivors discussed fears surrounding immigration status for themselves or family members who accompanied them to the hospital. Black survivors described challenges associated with role conflict, that is, prioritizing their roles as mothers, spouses, and as someone who was always “doing for somebody else.” Role conflict has been well described [37], and the tendency to prioritize others before oneself, concerns for children (e.g., surviving for the sake of the children) are commonly described among racial minority survivors [38,39,40,41].

Notably, these data were collected during the COVID-19 pandemic—a global event that has significantly influenced all aspects of daily life, including access to and receipt of timely healthcare. Changes to cancer care in the U.S. included discontinuing cancer screenings, delaying cancer surgeries, and reducing or delaying treatment to reduce the risk of COVID-19 infection for cancer populations who may be immunocompromised [42,43,44]. Even within this context, the Acceptability domain of HCA (quality of patient–provider interaction) was the most salient domain across all racial groups, highlighting the importance of empathy, compassion, and clear communication from providers to ensure high-quality care.

The Acceptability and Accommodation domains demonstrated strong psychometric properties, with component patterns having moderate to high item loadings, and importantly, meeting or exceeding established cut points for reliability. The final model structures also demonstrated good model fit, meeting or exceeding values considered as evidence of good fit. The internal reliability estimates for each of the sub-dimensions also indicate that the items within each dimension relate to each other and support the interpretation that the item sets measure similar underlying constructs, and high composite reliability and AVE for both domains indicate the internal consistency of scale items. Although AVE was low for ‘Access to support services’, it is still acceptable given the high composite reliability, as explained by Fornell and Larcker [23]. Additionally, the weak correlations observed for Accommodation indicate that there may be two distinct sub-domains of this dimension, whereas high correlations observed for Acceptability indicate all the sub-domains are a part of same latent construct. This study makes an important contribution to the field and evaluates the relevance of the Penchansky and Thomas framework [3] to contemporary and diverse cancer survivor populations. Relative to other qualitative analyses, our focus group sample was larger and included participants from diverse racial backgrounds to understand how these demographic factors may impact HCA and treatment. The framing of the focus group discussions and qualitative coding around the pre-defined construct of HCA provided a framework to guide the discussion and add context that was directly relevant to the topic, but it remained flexible enough to capture other emergent codes. One focus group was facilitated by a native Spanish speaker, enabling us to capture rich information regarding the experience of Hispanic survivors, which is a key limitation in previous studies. Although our current study included only English measures, subsequent analysis will determine measurement equivalence for Spanish measures as more data become available. The strengths of our psychometric evaluation include the use of multimodal data collection through paper, electronic and telephonic methods to administer the survey.

Several limitations should be noted. Survivors in the focus groups had varying treatment experiences, including differences in cancer stage and time since diagnosis. While this provided us with a range of experiences (e.g., currently receiving treatment vs. 10 years post-treatment), we were unable to determine how time since treatment impacted survivors’ perceptions of HCA. Furthermore, survivors who completed treatment many years prior to the focus group may have been vulnerable to recall bias. However, one of the questions assessed by facilitators was “How well do you remember your cancer treatment experience,” and participants noted that their cancer treatment was such a life-altering experience, they were able to remember even the most trivial details. Additionally, as a means of protecting privacy, we did not assign statements to specific individuals, so we do not have quantifiable data on HCA mentions by race. The limitations of the psychometric evaluation include its inclusion of only a few U.S. states and ovarian cancer survivors, limiting the generalizability of results to the broader U.S., to females with other cancer types, and to male cancer survivors. We did not test for other types of validity, and hence, additional testing of the measures is needed to provide more evidence regarding construct validity. However, with an acceptable factor structure and reliability, these multi-item scales are promising instruments to be used in future studies that evaluate the Acceptability and Accommodation dimensions of HCA.

## 5. Conclusions

In conclusion, cancer survivors’ perceptions of HCA largely aligned with the framework proposed by Penchansky and Thomas, indicating that this framework captures important aspects of access in this population. Psychometric evaluation supports standardized measures of Accommodation and Acceptability using self-reported survey items, which will contribute to the better characterization of HCA dimensions among diverse cancer survivors. Improved rigor in studies examining healthcare access will provide a better understanding of care delivery patterns and disparities in care and will help guide interventions to provide equitable access to timely and quality cancer care.

## Figures and Tables

**Table 1:** Operational Definitions for Healthcare Access Dimensions and Emergent Codes.

HCA Dimension	Operational Definition ^1^	Examples
Acceptability	Patient’s attitude to personal and practice characteristics of healthcare provider	Empathy; compassion; provider’s respect for faith and beliefs; patient–provider communication
Accessibility	The physical location of medical professionals and treatment(s) in relation to the patient	Location and distance; transportation available; convenience of parking
Accommodation	Organization of healthcare resources in relation to patients’ convenience and ability to accommodate such services	Hospital/clinic schedule; wait times; ease of scheduling/rescheduling; language Accessibility/interpreter
Affordability	Pricing, willingness, and ability to pay for treatment and other forms of supportive and/or follow-up care	Income; insurance; insurance co-pays; missed hours of work/pay or forced to quit job
Availability	Type, quality, and volume of healthcare services in need relation to patient need	Number of doctors/hospitals, provider specialty and training; hospital/provider volume
**Emergent Code**	**Operational Definition ^2^**	**Examples**
Support	Factor or characteristic that supports one’s treatment journey	Attitude; faith; self-advocacy; support system
Challenges	Factor or characteristic that negatively impacts one’s treatment journey	Fear, inadequate support system, mental and emotional wellness, role conflict

^1^ Operational definitions for HCA dimensions are as described by Penchansky and Thomas. ^2^ Emergent code operational definitions are based on commonly accepted definitions.

**Table 2:** Codes for Healthcare Access Framework, Facilitators, and Barriers to Recovery.

	# Groups Mentioned ^1^	# Total Mentions ^2^	% Total Mentions
**HCA Dimension**			
Acceptability	7	108	41%
Accessibility	5	28	11%
Accommodation	7	54	20%
Affordability	7	37	14%
Availability	7	38	14%
**Facilitators**			
Attitude	5	5	5%
Faith	6	31	28%
Self-advocacy	4	9	8%
Support system	7	65	59%
**Barriers**			
Attitude	2	12	12%
Fear	7	46	46%
Inadequate support system	5	15	15%
Mental and emotional awareness	4	18	18%
Role conflict	4	9	9%

^1^ Groups mentioned refers to the number of focus group that mentioned each of the codes. ^2^ Total mentions describe the code frequency after reviewing participant statements.

**Table 3:** Goodness-of-fit Statistics for the Accommodation and Acceptability Dimensions.

Fit Statistic	Hypothesized Model Structures		
Accommodation	1-Factor First-Order Model	3-Factor First-Order Model	2-Factor First-Order Model
χ^2^	273.091 *	92.687	77.452
Df	65	74	64
*P*	0.000	0.070	0.120
RMSEA	0.098	0.028	0.025
CFI	0.780	0.980	0.986
TLI	0.736	0.976	0.983
SRMR	0.142	0.080	0.082
Acceptability	1-Factor First-Order Model	5-Factor First-Order Model	5-Factor Higher-Order Model
χ^2^	3536.770 *	1124.280 *	1119.895 *
Df	350	340	346
*P*	0.0000	0.0000	0.0000
RMSEA	0.166	0.083	0.082
CFI	0.825	0.957	0.958
TLI	0.812	0.952	0.954
SRMR	0.218	0.087	0.093

Note. All values have been rounded to the second decimal place for ease of presentation. RMSEA = root mean square error of approximation, CFI = comparative fit index, TLI = Tucker–Lewis index, Standardized Root Mean Square Residual (SRMR), * Indicates χ^2^ are statistically significant at *p* < 0.05. Values of 0.95 or greater for TLI and CFI and values < 0.08 for RMSEA have been taken as evidence of good fitting models (Hu and Bentler, 1999).

**Table 4:** Composite Reliability and Average Variance Extracted for Latent Constructs.

Multi-Item Scales (# of Items)	Latent Factor’s Composite Reliability (Ω)	Latent Factor’s Average Variance Extracted (AVE)
**Accommodation (# items: 14)**		
Satisfaction with care (# items: 5)	0.80	0.51
Access to support services (# items: 9)	0.82	0.36
**Acceptability (# items: 28)**	0.89	0.65
Trust (# items: 11)	0.93	0.56
Care for emotions (# items: 4)	0.94	0.79
Cultural competence (# items: 4)	0.91	0.71
Sharing information (# items: 6)	0.94	0.74
Other staff (# items: 3)	0.92	0.80

Note: an ideal reliability estimate for a multi-item scale should be > 0.70 and average variance extracted > 0.50.

## Data Availability

The data that support the findings of the study are available from the corresponding author upon reasonable request.

## Supplementary Materials

The following supporting information can be downloaded at: https://www.mdpi.com/article/10.3390/cancers14246266/s1, Supplemental Table S1: Item-Level description, Supplemental Table S2: Cohort Descriptive Statistics, Supplemental Table S3: Illustrative Quotes, Supplemental Table S4: Correlations with confidence intervals
