# Supplementary material for: Development and Psychometric Evaluation of Healthcare Access Measures among Women with Ovarian Cancer
Source: Cancers (Basel). 2022 Dec 19;14(24):6266. doi: 10.3390/cancers14246266 (PMC9776822; doi:10.3390/cancers14246266)
Supplement: Supplementary file 1 [file cancers-14-06266-s001.zip › cancers-2007601-supplementary.pdf]

**Supplemental Table S1: Item-Level description**				
**Accommodation Dimension:** Organization of healthcare services and resources in relation to patients' ability to accommodate such services				
**Item No.**	**Source**	**Factors and number of items**	**Question**	**Response Options**
1	Ambulatory Care Experiences Survey (ACES)[10]	Seeing same doctor repeatedly<br># of items 1	In the last 12 months, when you went to your doctor's office, how often did you see your primary ovarian cancer doctor (not an assistant or partner)? Would you say:	Always, Almost Always, Most of the time, Sometimes, Almost never, Never
2		Convenience of appointment<br># of items 1	In the last 12 months, how often were you able to get appointments at a date and time that was convenient for you? Would you say:	
3	Medicare Current Beneficiary Survey (MCBS)[11]	Satisfaction with various aspects of care<br># of items 3	**How satisfied or dissatisfied you have been with:**<br>The Availability of care at night and on weekends. Would you say:	Very satisfied, Satisfied, Dissatisfied, Very dissatisfied, Not applicable
4			The ability to contact your primary ovarian cancer doctor to get medical advice when you need it. Would you say:	
5			The ease of rescheduling appointments with your primary ovarian cancer doctor when you need it. Would you say:	
6_a	New Item	Access to support services<br># of items 9	**Some clinics have additional services available to assist patients during their treatment. At any time during or after your ovarian cancer treatment, did you need help, if yes, did you receive help with any of the following services:**<br>Free shuttle or van transportation	Yes, No

6_b			Free parking or parking vouchers	
6_c			Patient guides, patient navigators or social workers	
6_d			Financial services or copayment assistance	
6_e			Nutrition or exercise counseling or classes	
6_f			Symptoms management or physical therapy	
6_g			Cancer support group	
6_h			Childcare or eldercare services	
6_i			Mental health or psychological counseling	
**Acceptability Dimension:** Patients attitude to personal and practice characteristics of healthcare provider				
**Item No.**	**Source**	**Factors and number of items**	**Question**	**Response Options**
7	Medical Mistrust Scale[12]	Trust<br># of items: 11	**Now I would like to ask you some questions about your interactions and relationship with doctors. Please tell me how much you agree with the following statements:**<br>I cannot trust doctors and health care workers	Strongly agree, Agree, Disagree, Strongly disagree
8			I should be suspicious of information from doctors and health care workers.	
9			I should not confide in doctors and health care workers because it will be used against me	
10			Doctors and health care workers treat patients like guinea pigs	
11			I receive the same medical care from doctors and health care workers as people from other ethnic groups	
12			I am treated the same as people of other ethnic groups by doctors and health care workers	

13			In most hospitals, people of different ethnic groups receive the same kind of care	
14			Doctors have my best interests in mind	
15			Doctors and health care workers sometimes hide information from me	
16			I have personally been treated poorly or unfairly by doctors or health care workers because of my ethnicity	
17			I should be suspicious of modern medicine	
18	Patient-Centered Communication in Cancer Care (PCC-Ca)-36[13]	Care for emotions, # of items: 4	**How much did your doctor:** Pay attention to how you are doing emotionally	Not at all, Not very much, Somewhat, A lot, A great deal
9			Show concern for your feelings	
20			Show concern for how your family is doing emotionally?	
21			Make you feel comfortable to talk about your fears, stress and other feelings?	
22	New Item	Cultural Competence, # of item 1	How much did your doctor treat you with respect?	Not at all, Not very much, Somewhat, A lot, A great deal
23	Consumer Assessment of Healthcare Providers & Systems (CAPHS)[14]	Cultural Competence, # of items 3	**During the course of your treatment, how often did your doctor:** Ignore what you told him or her?	Never, Rarely, Sometimes, Often, Always
24			Interrupt you when you were talking?	
25			Use a condescending, sarcastic or rude tone or manner with you?	
26	Patient-Centered Communication in Cancer Care	Sharing Information, # of items 6	Make you feel comfortable asking questions?	Never, Rarely, Sometimes, Often, Always
27			Talk with you about your concerns and questions?	
28			Giver you helpful Information, even when you don’t ask for it?	

29	(PCC-Ca)-36[13]	How much did your doctor:	Make sure you have the information you need?	
30			Help you understand the information you need to know?	
31			Make sure your questions are answered?	
32	New Items	Other staff at doctor's office<br># of items 3	How often did nurses at your primary ovarian cancer doctor's office show that they care about you and your wellbeing?	Never, Rarely, Sometimes, Often, Always
33			How often did other staff at your primary ovarian cancer doctor's office show that they care about you and your wellbeing?	
34			How often did other staff at your primary ovarian cancer doctor's office treat you with respect?	

1

Supplemental Table S2: Cohort Descriptive Statistics

Patient Characteristics	N (%)
Total N	333
Median Age (years) at Diagnosis (IQR)	60 (46-66)
Patient Race	
White	272 (81.7)
Black/African American	45 (13.5)
American Indian or Alaska Native	2 (0.6)
Asian	2 (0.6)
Native Hawaiian or Pacific Islander	1 (0.3)
Other	9 (2.7)
Don't Know/Refuse	2 (0.6)
Education	
< HS	6 (1.8)
HS Graduate or GED	60 (18%)
Some college or 2 year degree	90 (27%)
4 year college graduate	87 (26%)
> 4 year college graduate	93 (28%)

**Supplemental Table S3. Illustrative Quotes**
**HCA Dimensions**
**Dimension 1: Acceptability**
And when I got the diagnosis, I told the doctor. He began to explain to me what I was gonna have to go through as far as my treatments was concerned. And my concern to him was, "how successful is the treatment that you're giving to me?" "How successful has it been in our Black community?" Because I know and I feel that sometimes the treatments that they give to other people sometimes they're not the same treatment that work very well for us. And we talked about that. And it was- I really felt good. They made me feel good about the treatment that I was receiving. It made me feel good about how it would work for me. They explained how it worked in the Black community.
When I first went in all my doctors was so lovely, and nurses, and they still are. But I had one lady doctor to come in and she just blurted stuff out at me and I just looked at her. And I couldn't understand anything that she said to me. I mean she was English, but the way she bottled it all up. And she said it in a hateful tone not in a nice tone or anything. And my daughter came back in she said, "What's wrong?" I said, "That doctor."
There's a saying we have: we don't care what you know until you show us that you care.
It doesn't matter to me as long as you respect me. You're going to- 'Cause really, I really think the discussion about caring really just means that you're feeling that if someone cares about me they're going to give me their best work. And that's the bottom line, you want the best work. And if you care about me you're going to give me your best. I think there's a relationship between do they care, do they have good bedside manners? You relate that to getting quality care.
**Dimension 2: Accommodation**
I am hearing I will be sick with nausea, and with all the appointment being told someone has to be with you. Who is going to take me there, being single?. Can't have people losing paychecks to take me to an appointment. I had to find someone to help, and that person made the appointment at a time that they were available. This delayed treatment. I was told if I showed up alone I would not get treated. Someone had to check in with me and stay for the entire time, they couldn't drop off and pick up
**Dimension 3: Availability**

I think one of the biggies is GYN oncology when you have ovarian cancer. Like, a gynecologist is not really capable of treating this correctly. So, if you live in a smaller town you don't have access to GYN ONC. I mean, we live in a pretty good-sized town and we're fortunate to have quite a few here. But, I'm a sales rep with a huge territory and everywhere I go in Kentucky, like, there's not GYN ONC anywhere, you know, other than here and, like, Lexington, so. So, I think that's something that with ovarian cancer in particular is something that's really necessary for access to quality care is access to the GYN ONC specialists.

Dimension 4: Affordability

Our family went down to one income because of my diagnosis and I could not help physically at home. First year was hardest, being unemployed and sick a lot from chemo.

I had to apply for financial support. Even though I had a job, I still needed help with the copays. Coworkers donated vacation time, was able to take laptop with me during treatment so I didn't have to use my vacation time

Dimension 5: Accessibility

We have a Department of Transportation that's called CAT. The challenge is, for some, if their appointment is at two o'clock and somebody else's appointment is at seven o'clock in the morning, they have to leave on transportation in the morning at seven, go to wherever they are to wait for the two o'clock appointment. And if they have someone whose appointment is not until four or five, so it's all day for them. So that has been disappointing to some people. So we are still trying to come up with better means of getting people to wherever they are going for transportation so that they will not have to depend on CDOT.

Facilitators and Barriers

Facilitators

Support System

Our support group was a very, very good place to come for support, encouragement, motivation, and things of that nature. And I did not have a number of a support place to call online. I did not want to do that. I preferred to talk to people that had related with- actually my experience.

I was going to say, it does help to talk about it…when we talk about it, it helps us minister to other people and it makes it much easier for us. It seems like even though we went through all of that it still makes it easier on us. We can kind of identify with what they're going through and help somebody or encourage somebody and let them know that they can make it or they can hold on - you know, just hold on.

Throughout all the treatment I've always gotten the copies of my MRI. Even though I don't understand every other word on the MRI, I Google everything and I talk to my mom and I talk to

	my brother, who are medical professionals too, to make sure I have full understanding of what's going on.
Faith	
	I'm three years survivor and I can remember my journey really [unclear] And I'm still going through it. I thank God for giving me the strength to go through it and that I was able to help someone else. 'Cause I get a lot of calls and I'm able to talk to a lot of people and … I can say I'm still going through this journey.
Positive Attitude	
	And that was a very, very important factor to me – to have doctors that didn't just push me through like I was a number. That sit on the bed beside me and held my hand and talked to me and encouraged me to be strong because they said I had a lovely attitude. And they helped me to continue to see that because that – my attitude – determined a lot about how I got through my process.
**Barriers**	
Fear	
	I think my barriers were mental. And, you know, going into it thinking I was going to die because that's what you saw on TV.
	I- I mean it was a very good experience, so, and I guess I was- If I had to sit there, I guess three or four hours, I was still going to sit there 'cause I was really frightened at the time. And I'm still frightened that it might come back even after nineteen years.
	And I have seen, as I am, I have noticed for example in Lincoln, I am on the board of directors and I know many people from the community who are afraid to go, for example, to the Lincoln hospital where they will receive everything for free. They will not be charged a penny for any exam. But one of the reasons that some are afraid of, is to be asked for the papers, that they will be reported, even if they are residents. But they go with someone who is not a resident who is not documented and they have problems, they are afraid that they will report them to the government.
	I just think as a people we might not have the health insurances- we might not have the jobs that have the health insurances to be able to cover these things, so I think, as I said, people won't approach it because they don't know how they're going to make their ends meet. You know, so the fear of the unknown. I mean, I think that's culturally, people just- some people just will not go to the doctor. You have to kind of hogtie them to make them even go see what something is and by the time they do in some cases it's too late
Mental and Emotional Awareness	

	For me it was also a shock. I was afraid. I am the typical Latino and I mean that we are afraid that we will be tested after being 40 years of age because we are Latino. We are machos, although I was not raised that way. But the fear, that I, I don't know, I thought, I knew, that I was afraid that they were going to tell me that I had cancer. And evidently the two doctors, my urologist and another oncologist, good specialists at the University of the South, at the medical school, my personal doctor said to me, well, let's do an operation. And that's where the depression came from for me.
	And I heard her, but I didn't hear her 'cause it's hard to explain just it's kind of like your brain is in a fog as your trying to take in this information. And you're still in denial that you even have cancer.
Role Conflict	
	One thing that I guess maybe prevented me from joining a support group was the fact that I was a single mother. And so, I was just like, "I can't- I don't have time to go to these [unclear]. I have to pay a babysitter." Why am I going to pay a babysitter so I can go boohoo with a bunch of women? Like, I need somebody to come to my house and put my three-year-old down to bed so that I can go to bed, you know? And so I did not- I mean I had my chemo buddy and that was cool. But it wasn't until I had finished treatment and I found Little Pink Houses of hope and went on a week-long retreat. That was the first time I was able to sit down with a group of women who had gone through something similar.
	I didn't want people to just- 'Cause I was so used to doing for everybody else. I'm always doing something for everybody else. When it was my time for somebody to do for me I wouldn't let that happen, I didn't want that. So, I had to learn to get somebody, to allow people to help me through everything I was going through. I had to allow them to help me. But it was hard.

Supplemental Table S4: Correlations with confidence intervals

Variable	1	2	3	4	5	6	7
Accommodation							
1. Satisfaction with care							
2. Access to support services	.44**<br>[.30, .56]						
Acceptability							
3. Trust	.27**<br>[.13, .40]	.22**<br>[.10, .33]					
4. Care for emotions	.50**<br>[.37, .61]	.22**<br>[.11, .32]	.32**<br>[.18, .45]				
5. Culture competence	.56**<br>[.44, .66]	.26**<br>[.12, .39]	.40**<br>[.26, .52]	.94**<br>[.92, .96]			
6. Sharing information	.57**<br>[.44, .67]	.24**<br>[.11, .37]	.36**<br>[.21, .48]	.92**<br>[.88, .94]	.95**<br>[.94, .97]		
7. Other staff	.54**<br>[.41, .64]	.22**<br>[.10, .35]	.34**<br>[.20, .47]	.69**<br>[.60, .77]	.76**<br>[.68, .82]	.75**<br>[.67, .81]	
8. Acceptability (overall latent construct)	.56**<br>[.44, .66]	.25**<br>[.11, .38]	.39**<br>[.25, .52]	.92**<br>[.96, .98]	.99**<br>[.99, .99]	.98**<br>[.97, .98]	.77**<br>[.70, .83]

Note. Values in square brackets indicate the 95% confidence interval for each correlation.

* indicates $p < .05$. ** indicates $p < .01$.
